# Advances in Brain–Computer Interfaces (BCI): Challenges and Opportunities

**DOI:** 10.3390/biomimetics11020157

**Published:** 2026-02-22

**Authors:** Yuchun Wang, Minyan Ge, Shumao Xu

**Affiliations:** 1 Institute of Science and Technology for Brain-Inspired Intelligence (ISTBI), Fudan University, Shanghai 200433, China; 2 Huck Institute of Life Science, The Pennsylvania State University, University Park, PA 16802, USA

It appears that the frontier of neural engineering is rapidly advancing towards seamless integration between biological neural networks and digital systems. This evolution reflects the growing consensus that Brain–Computer Interfaces (BCIs) are no longer merely experimental tools for signal translation, but dynamic systems capable of mimicking and augmenting the brain’s intrinsic information-processing mechanisms. As we bridge the brain to the world beyond healthcare [1], the field is witnessing a convergence of materials science, deep learning, and clinical rehabilitation.

To fully realize the potential of BCIs, recent research has emphasized the importance of foundational hardware and physiological understanding. Innovations in optoelectronics [2] and injectable fluorescent neural interfaces [3] are providing unprecedented resolution for monitoring and modulating neural activity. Concurrently, the development of robust physical interfaces, such as laser-patterned copper electrodes for personal healthcare [4] and injectable ultrasonic metagels for intracranial monitoring [5], is solving critical challenges in biocompatibility and signal fidelity.

Beyond recording, the therapeutic scope of BCI is expanding through precise modulation techniques. Novel approaches like non-invasive temporal interference stimulation are enabling precision at the deep brain level [6], while emerging imaging techniques using bioluminescence molecules promise to transform how we visualize these deep structures [7]. Furthermore, a holistic understanding of neural health, considering heart–brain connections [8], metabolic reprogramming in ischemic stroke [9], and brain–body interactions involving vagus nerve stimulation [10], is proving essential for effective intervention.

This broad spectrum of innovation sets the stage for clinical translation, particularly in stroke and neurodegenerative diseases. Recent breakthroughs demonstrate that long-term BCI functional electrical stimulation can significantly enhance neuroplasticity and functional recovery in elderly stroke patients [11]. Portable adaptive solutions, such as the Magnetic NeuroRing for real-time transcranial magnetic stimulation [12], are making home-based rehabilitation a reality. Moreover, deep learning is closing the gap in silent stroke screening by utilizing retinal data [13], and specialized interfaces are facilitating communication for ALS patients [14].

Against this backdrop of rapid technological and clinical progress, this Special Issue, “Advances in Brain–Computer Interfaces (BCI): Challenges and Opportunities”, brings together original research articles and one comprehensive review paper. These contributions delve into specific challenges within this landscape, covering signal classification, affective computing, sleep staging, and preclinical disease detection.

A principal focus of this issue is the enhancement of signal decoding algorithms to handle the non-stationary nature of EEG data. In their work, Lee et al. [15] address the critical challenge of signal quality in practical BCI applications, proposing a multi-window ensemble model that mimics the brain’s ability to integrate temporal information, which demonstrates superior performance in decoding motor intentions even under conditions of signal degradation. This robustness is essential for moving BCI technology from controlled laboratory settings to real-world environments.

Building on the theme of advanced classification, Zhang et al. [16] explore the use of transfer learning to overcome the challenge of individual variability in EEG signals. The authors present a method that leverages knowledge from related domains to improve classification accuracy across different subjects, addressing one of the major bottlenecks in user-independent BCI calibration.

Beyond motor control, this issue highlights the growing role of BCIs in affective computing and environmental interaction. Addressing this, Ma et al. [17] introduce “MSBiLSTM-Attention: EEG Emotion Recognition Model Based on Spatiotemporal Feature Fusion”. By integrating multi-scale features with attention mechanisms, their model effectively captures the complex spatiotemporal dynamics of human emotion, paving the way for systems that can recognize and respond to a user’s psychological state.

Complementing this, Li et al. [18] investigate the external feedback loop in “Designing Light for Emotion: A Neurophysiological Approach to Modeling Affective Responses to the Interplay of Color and Illuminance”. Their findings provide a neurophysiological basis for intelligent lighting systems that dynamically regulate user emotion, exemplifying a closed-loop interaction between the environment and the brain.

The application of BCI technology in clinical diagnostics and health monitoring is another vital theme. Wang et al.’s [19] contribution, “MASleepNet: A Sleep Staging Model Integrating Multi-Scale Convolution and Attention Mechanisms”, addresses the increasing prevalence of sleep disorders by offering a highly accurate, automated method for sleep staging, which is crucial for the diagnosis and management of sleep-related pathologies. Similarly, in the realm of neurodegenerative disease, Li et al. [20] propose “Task-Related EEG as a Biomarker for Preclinical Alzheimer’s Disease: An Explainable Deep Learning Approach”. Their study demonstrates that task-related EEG, combined with interpretable deep learning, can serve as an effective screening tool for early-stage Alzheimer’s, offering a non-invasive window into neural cognitive decline.

Finally, this volume features a narrative review that synthesizes the clinical impact of these technologies. Ortega-Robles et al. [21], in “Brain–Computer Interfaces in Parkinson’s Disease Rehabilitation”, provide a comprehensive overview of how eBCIs are being utilized to address both motor and non-motor symptoms of Parkinson’s disease. The review highlights the shift towards personalized neurorehabilitation, identifying current limitations and outlining future directions for therapeutic BCI applications.

Across these thematic strands, the contributions of this Special Issue collectively redefine the scope of BCIs. From robust decoding algorithms and emotion-aware systems to diagnostic tools and rehabilitation therapies, these studies illustrate that BCI technology is evolving from simple signal translation to complex, bidirectional symbiosis between human and machine. This collection aims to serve as a reference for future interdisciplinary research, fostering collaboration among neuroscientists, engineers, clinicians, and data scientists.

The Guest Editors of this Issue extend their sincere gratitude to all contributing authors for their innovative research, to the reviewers for their thoughtful evaluations, and to the *Biomimetics* editorial team for their support and professionalism.

## References

[1] Xu S., Liu Y., Lee H., Li W. (2024). Neural interfaces: Bridging the brain to the world beyond healthcare. Exploration.

[2] Xu S., Momin M., Ahmed S., Hossain A., Veeramuthu L., Pandiyan A., Kuo C.C., Zhou T. (2023). Illuminating the brain: Advances and perspectives in optoelectronics for neural activity monitoring and modulation. Adv. Mater..

[3] Xu S., Xiao X., Manshaii F., Chen J. (2024). Injectable fluorescent neural interfaces for cell-specific stimulating and imaging. Nano Lett..

[4] Xu S., Ge M., Chen S., Wang Y., Tang Y., Wang J., Cui X., Sun C., Zeng H., Wang N. (2025). Leveraging Laser-Patterned Copper Electrodes for Personal Healthcare. Small Methods.

[5] Wang Y., Ge M., Wang R., Zhu Y., Xu S. (2025). Injectable ultrasonic metagels for intracranial monitoring. npj Biosens..

[6] Xu S., Cui H., Xiao X., Manshaii F., Hong G., Chen J. (2025). Precision at Deep Brain: Noninvasive Temporal Interference Stimulation. ACS Nano.

[7] Xu S., Manshaii F., Chen J. (2024). Is deep brain imaging on the brink of transformation with a bioluminescence molecule?. BMEMat.

[8] Xu S., Scott K., Manshaii F., Chen J. (2024). Heart-brain connection: How can heartbeats shape our minds?. Matter.

[9] Wang Y., Ge M., Wang J., Xu Y., Wang N., Xu S. (2025). Metabolic reprogramming in ischemic stroke: When glycolytic overdrive meets lipid storm. Cell Death Dis..

[10] Wang Y., Yang Z., Wang J., Ge M., Wang N., Xu S. (2025). Brain-Body Interactions in Ischemic Stroke: VNS Reprograms Microglia and FNS Enhances Cerebellar Neuroprotection. Stroke.

[11] Chen S., Xie N., Tang Y., Ji Y., He Z., Wang Y., Huang X., Fu J., Ge M., Liu Q. (2025). Long-Term Brain–Computer Interface Functional Electrical Stimulation Enhances Neuroplasticity and Functional Recovery in Elderly Stroke: A 4.5-Year Longitudinal Study Integrating Electroencephalography Biomarkers and Clinical Assessments. Research.

[12] Tang Y., Wang Y., Zhang W., Liu X., Li Y., Hu W., Ding L., Feng F., Chen X., Feng J. (2026). Magnetic NeuroRing: A portable adaptive brain-computer interface for real-time transcranial magnetic stimulation in post-stroke motor rehabilitation. npj Biomed. Innov..

[13] Ge M., Wang Y., Xu S. (2025). From retina to brain: How deep learning closes the gap in silent stroke screening. npj Digit. Med..

[14] Wang Y., Tang Y., Wang Q., Ge M., Wang J., Cui X., Wang N., Bao Z., Chen S., Wang J. (2025). Advances in brain computer interface for amyotrophic lateral sclerosis communication. Brain-X.

[15] Lee D.-G., Lee S.-B. (2025). Robust Motor Imagery–Brain–Computer Interface Classification in Signal Degradation: A Multi-Window Ensemble Approach. Biomimetics.

[16] Zhang W., Tang X., Dang X., Wang M. (2025). A Capsule Decision Neural Network Based on Transfer Learning for EEG Signal Classification. Biomimetics.

[17] Ma Y., Huang Z., Yang Y., Chen Z., Dong Q., Zhang S., Li Y. (2025). MSBiLSTM-Attention: EEG Emotion Recognition Model Based on Spatiotemporal Feature Fusion. Biomimetics.

[18] Li X., Wang R., Whang M. (2025). Designing light for emotion: A neurophysiological approach to modeling affective responses to the interplay of color and illuminance. Biomimetics.

[19] Wang Z., Gong Z., Wang T., Dong Q., Huang Z., Zhang S., Ma Y. (2025). MASleepNet: A Sleep Staging Model Integrating Multi-Scale Convolution and Attention Mechanisms. Biomimetics.

[20] Li Z., Wang H., Li L. (2025). Task-Related EEG as a Biomarker for Preclinical Alzheimer’s Disease: An Explainable Deep Learning Approach. Biomimetics.

[21] Ortega-Robles E., Carino-Escobar R.I., Cantillo-Negrete J., Arias-Carrión O. (2025). Brain–Computer Interfaces in Parkinson’s Disease Rehabilitation. Biomimetics.

